# Retraction: Fischer’s ratio and DNA damage in hypoxemia-induced brain injury in rat model: Prophylactic role of quercetin and mexamine supplementation

**DOI:** 10.1371/journal.pone.0343460

**Published:** 2026-02-23

**Authors:** The PLOS One Editors

After this article [1] was published, concerns were raised about the comet assay in Fig 3.

Specifically, regions of the individual comet tails in the different panels of Fig 3 appear similar to each other.

Upon editorial follow-up, the corresponding author stated that these images are merely illustrative to show the extent of the DNA damage and that the comet assay data were provided by a third party after the experiments were conducted at a different laboratory.

The corresponding author provided the underlying images used to create Fig 3 and the underlying quantitative comet assay data. However, after editorial review, the *PLOS One* Editors consider that the underlying data provided do not resolve the issue and raise further concerns about the integrity and reliability of the reported results.

In light of the above issues, the *PLOS One* Editors retract this article.

MOK and HA did not agree with the retraction.
